# Dr. J. Miller Fothergill on the Use of Maltine

**Published:** 1881-12-20

**Authors:** 


**Dr. J. Miller Fothergill on the use of Maltine,** says, in London Practitioner: In order to aid the defective action upon starch by the natural diastase being deficient in quantity or impaired in power, we add the artificial diastase "maltine." But, as Dr. Roberts points out, in order to make this ferment operative it must not be taken after a meal is over. Rather it should be added to the various forms of milk porridge or puddings before they are taken into the mouth. About this there exists no difficulty. Maltine is a molasses-like matter and mixes readily with the milk, gruel, etc., without interfering either with its attractiveness, or its toothsomeness; indeed its sweet taste renders the gruel, etc., more palatable. A minute or two before the milky mess is placed before the child. or invalid, the maltine should be added. If a certain portion of baked flour, no matter in what concrete form, were added to plain milk, and some maltine mixed with it, before it is placed on the nursery table, we should hear much less of infantile indigestion and mal-nutrition.

